# Supplementary material for: Causal Relationship between Aging and Anorexia Nervosa: A White-Matter-Microstructure-Mediated Mendelian Randomization Analysis
Source: Biomedicines. 2024 Aug 16;12(8):1874. doi: 10.3390/biomedicines12081874 (PMC11351342; doi:10.3390/biomedicines12081874)
Supplement: Supplementary file 1 [file biomedicines-12-01874-s001.zip › Supplementary Figure/Figure S4/Singe SNP plot of MR analysis for TL to ubm-b-1913.pdf]

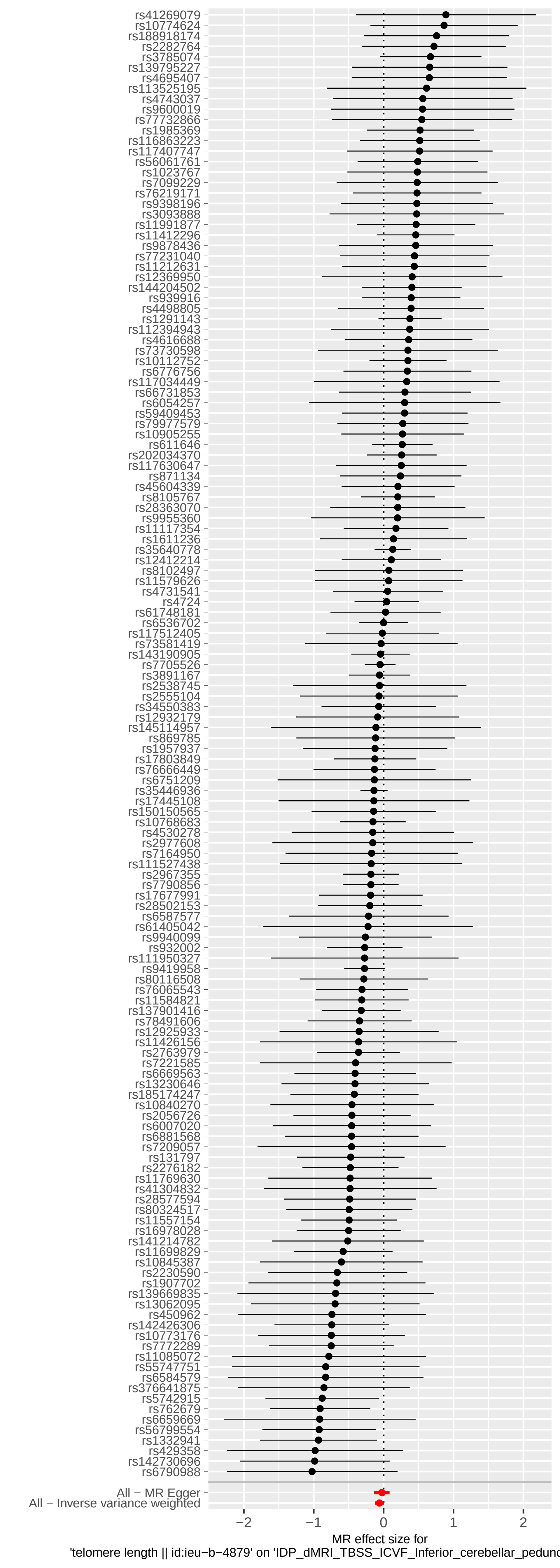
rs41269079
rs10774624
rs188918174
rs2282764
rs3785074
rs139795227
rs4695407
rs113525195
rs4743037
rs9600019
rs77732866
rs1985369
rs116863223
rs117407747
rs56061761
rs1023767
rs7099229
rs76219171
rs9398196
rs3093888
rs11991877
rs11412296
rs9878436
rs77231040
rs11212631
rs12369950
rs144204502
rs939916
rs4498805
rs1291143
rs112394943
rs4616688
rs73730598
rs10112752
rs6776756
rs117034449
rs66731853
rs6054257
rs59409453
rs79977579
rs10905255
rs611646
rs202034370
rs117630647
rs871134
rs45604339
rs8105767
rs28363070
rs9955360
rs11117354
rs1611236
rs35640778
rs12412214
rs8102497
rs11579626
rs4731541
rs4724
rs61748181
rs6536702
rs117512405
rs73581419
rs143190905
rs7705526
rs3891167
rs2538745
rs2555104
rs34550383
rs12932179
rs145114957
rs869785
rs1957937
rs17803849
rs76666449
rs6751209
rs35446936
rs17445108
rs150150565
rs10768683
rs4530278
rs2977608
rs7164950
rs111527438
rs2967355
rs7790856
rs17677991
rs28502153
rs6587577
rs61405042
rs9940099
rs932002
rs111950327
rs9419958
rs80116508
rs76065543
rs11584821
rs137901416
rs78491606
rs12925933
rs11426156
rs2763979
rs7221585
rs6669563
rs13230646
rs185174247
rs10840270
rs2056726
rs6007020
rs6881568
rs7209057
rs131797
rs2276182
rs11769630
rs41304832
rs28577594
rs80324517
rs11557154
rs16978028
rs141214782
rs11699829
rs10845387
rs2230590
rs1907702
rs139669835
rs13062095
rs450962
rs142426306
rs10773176
rs7772289
rs11085072
rs55747751
rs6584579
rs376641875
rs5742915
rs762679
rs6659669
rs56799554
rs1332941
rs429358
rs142730696
rs6790988
All − MR Egger
All − Inverse variance weighted
−2
−1
0
1
2
MR effect size for
'telomere length || id:ieu−b−4879' on 'IDP_dMRI_TBSS_ICVF_Inferior_cerebellar_pedunc
